# Honghua Xiaoyao tablet combined with estradiol improves ovarian function in D-galactose-induced aging mice by reducing apoptosis and affecting the release of reproductive hormones: an *in vivo* study

**DOI:** 10.3389/fphar.2024.1394941

**Published:** 2024-06-06

**Authors:** Chan Su, Ruihong Zhang, Xiujuan Zhang, Xiaoning Feng, Qiong Wu, Yiwei Gao, Jing Hao, Yu-lan Mu

**Affiliations:** ^1^ Department of Gynecology, Provincial Hospital, Affiliated to Shandong First Medical University, Jinan, China; ^2^ Department of Gynecology, Taiyuan Maternal and Child Health Hospital, Taiyuan, China; ^3^ Key Laboratory of Experimental Teratology, Ministry of Education, Department of Histology and Embryology, School of Medicine, Shandong University, Jinan, China; ^4^ The Second Clinical Medical College of Shandong University of Traditional Chinese Medicine, Jinan, China

**Keywords:** Honghua Xiaoyao tablet, aging mice, apoptosis, sex hormone, FSHR

## Abstract

**Context:** It is very necessary to delay ovarian aging and prevent age-related health problems. The active ingredient in Honghua Xiaoyao tablet (HHXYT) has the effects of anti-oxidation, anti-inflammation, immune regulation and so on.

**Objective:** To explore the effect and mechanism of Honghua Xiaoyao tablet on aging model mice.

**Materials and methods:** The aging model was established by intraperitoneal injection of D-galactose in model mice. The mice in the HHXYT-L,M,H group were given 0.3 g/kg, 0.6 g/kg and 1.2 g/kg Honghua Xiaoyao tablet suspension respectively, and the HHXYT-M + E2 group was given 0.6 g/kg HHXYT +0.13 mg/kg estradiol valerate for 30 days. In this study, ELISA, HE, Western blot, IH and TUNEL were used.

**Results:** HHXYT + E2 can improve the gonadal index, estrous cycle of aging mice. In HHXYT-M + E2 group, the level of FSH and LH decreased, while E2 and AMH increased significantly. The number of growing follicles in HHXYT-M + E2 group increased, which was better than that of HHXYT alone. Western blot results showed that HHXYT-M + E2 group decreased the expression of Bax, cleaved-Parp, cleaved-Casp-3 and CytC molecules and increased the expression of Bcl-2 in ovarian tissue. FSHR expression decreased in model group and increased in HHXYT group. TUNEL staining showed that the number of apoptotic cells in HHXYT group was reduced, and the HHXYT-M + E2 group was the most significantly.

**Discussion and conclusion:** HHXYT can improve the level of sex hormones and increase the number of growing follicles in aging mice. HHXYT-M + E2 group has the best effect, and its mechanism may be related to reducing ovarian granulosa cell apoptosis.

## 1 Introduction

Senescence is the gradual decline in cellular and bodily function, encompassing both physiological and pathological processes (Kubben and Misteli, 2017). Most countries in the world are facing the challenge of an aging population (Zeng et al., 2017). Aging and related diseases affect human health. Ovary, as a key female reproductive organ, is one of the organs showing early-onset aging-related dysfunction in human beings, which declines obviously only after 30 years old (Broekmans et al., 2009; Tilly and Sinclair, 2013). The fluctuation or decrease of sex hormones caused by ovarian aging can cause a series of physical and psychological symptoms. Current evidence suggests that factors such as age, smoking, high-sugar diet, stress, superovulation, chemotherapeutic drugs and industrial pollutants may accelerate ovarian aging by exacerbating oxidative stress (OS) (Yan et al., 2022). By slowing down the process of ovarian aging, preventing and reducing aging-related health problems, it is necessary to better understand the aging mechanism and develop effective anti-aging interventions.

In order to study the mechanism of aging, natural aging model and accelerated aging model are generally used. The natural aging model takes a long time and high cost, so the accelerated aging model is selected in this experiment. The accelerated aging process induced by D-galactose was highly similar to that of human aging, and the animal survival rate was higher during the experimental period (Azman and Zakaria, 2019). This model is widely used to explore the mechanism of ovarian senescence. Galactose toxicity delayed the onset of puberty in rats and formed a state of high gonadotropin and low estrogen (Bandyopadhyay et al., 2003). The treatment of mice with D-galactose leads to excessive production of ROS and advanced glycation end products (AGE) in multiple organs, including ovaries (Liang et al., 2020). The accumulation of ROS and AGE are the reasons for the aging of ovarian function (Semba et al., 2010; Tatone and Amicarelli, 2013), the accumulation of ROS leads to the increase of toxic metabolites, which mainly induce the apoptosis of ovarian granulosa cells and damage the development of follicles (Tatone and Amicarelli, 2013).

Traditional Chinese medicine has a unique theoretical basis and rich clinical experience in delaying aging (Mu-Hua et al., 2019). Honghua Xiaoyao tablet is composed of nine kinds of Chinese herbs such as Angelica sinensis and Radix Paeoniae Alba, which is used to treat chest pain caused by liver qi discomfort, irregular menstruation, breast pain and other discomfort. Mi et al. (2020) used the combination of UPLC-Q-TOF/MS and HPLC-QQQ/MS to comprehensively characterize the chemical composition and potential quality markers of HHXYT, and quantified 14 marker components in HHXYT. A total of 55 components were clearly characterized or preliminarily identified. Modern medical scholars have found that Angelica has the effects of promoting blood circulation and tonifying blood, anti-oxidation, anti-inflammation and analgesia (Peiliang et al., 2019). Paeoniflorin (Pae) and glycyrrhizin (Liq) are important chemical components of HHXYT, which have a wide range of anti-inflammatory and immunomodulatory effects (Xin et al., 2019; Zhang and Wei, 2020). The mechanism of drug anti-aging is multifaceted, and intervention measures are often aimed at several characteristics at the same time. At present, the relevant research is still lacking.

In this study, D-galactose induced aging mouse model was used to study the role of HHXYT combined with estradiol in the process of ovarian senescence. By detecting the level of serum sex hormones and the changes of ovarian FSH receptor (FSHR), the expression of apoptosis-related signal molecules in ovarian tissue was analyzed to clarify the possible mechanism of drug action, so as to provide scientific basis for the clinical research of HHXYT.

## 2 Materials and methods

### 2.1 Animals

A total of 60 8-week-old SPF grade female ICR mice, weighing 29–33 g and unmated, were purchased from Speyford Beijing Biotechnology Co., Ltd. The mice were raised separately in the SPF animal room of the central laboratory of the affiliated Provincial Hospital of Shandong First Medical University. The mice ingested food and water freely and kept regular light for 12 h. This experiment was approved by the Laboratory Animal Ethics Committee of the Provincial Hospital affiliated to Shandong First Medical University. The approval number is NO. 2020-031.

### 2.2 Drugs and reagents

D-galactose (500g/bottle), Shanghai McLean Biotechnology Co., Ltd., batch NO.C12049311; Honghua Xiaoyao tablet (0.39 g/tablet) (Table 1), Jiangxi Puzheng Pharmaceutical Co., Ltd., batch NO.Z20080299; Wright-Giemsa solution (Beijing Solebao Biotechnology Co., Ltd., batch NO.418031); Hematoxylin-eosin solution (Beijing Solebao Biotechnology Co., Ltd., batch NO.G1080, G1100). Follicle stimulating hormone (FSH) enzyme-linked immunosorbent assay (ELISA) kit (Shanghai Langton Biotechnology Co., Ltd., batch NO.BPE20419), luteinizing hormone (LH), estradiol (E2), anti-mullerian hormone (AMH) ELISA kit (Boyan Biotechnology Co., Ltd., respectively batch NO.BY-R221468, BY-JZF0048, BY-R20514). FSHR Antibody (ELK Biotechnology), Bcl-2-associated X protein (Bax) Antibody, B-cell lymphoma-2 (Bcl-2) Antibody (Affinity), poly-ADP ribose polymerase (Parp) Antibody, cleaved-Parp Antibody, caspase-3 (casp-3) Antibody, cytochrome C (CytC) Antibody (Abmart), cleaved-Casp-3 Antibody (Abcam), Western second Antibody, IgG-HRP second Antibody (Abmart), PVDF membrane (Millipore, Germany), Western Chemiluminescence solution A/B (Bioexplorer); DAB stain (ZSGB-BIO); TUNEL apoptosis detection kit (Elabscience, batch NO.E-CK-A320).

**TABLE 1:** The compositions of Honghua Xiaoyao tablet.

No.	Chinese name	Latin name	Contents(g)/1000 pills
1	Danggui	*Angelica sinensis (Oliv.) Diels*	260
2	Baishao	*Paeonia lactiflora Pall*	260
3	Baizhu	*Atractylodes macrocephala Koidz*	260
4	Fuling	*Poria cocos(Schw.) Wolf*	260
5	Honghua	*Carthamus tinctorius L*	50
6	Zaojiaoci	*Gleditsia sinensis Lam*	80
7	Zhuye Chaihu	*Bupleurum marginatum Wall. ex DC*	260
8	Bohe	*Mentha haplocalyx Briq*	40
9	Gancao	*Glycyrrhiza uralensis Fisch*	195

Note: The pharmaceutical ingredients, content and production method of Honghua Xiaoyao tablet are provided by the manufacturer of Jiangxi Puzheng Pharmaceutical. The names of the plants are consistent with the names of the http://www.worldfloraonline.org website (website access time: 2023/12/03 Beijing time).

### 2.3 Equipment and instruments

Pipette Gun (RAININ, USA); Ultracentrifuge (Thermo, USA); Analytical balance (Ohaus, USA); Enzyme labeling instrument (Tecan, Switzerland); Paraffin embedding Machine and Paraffin slicer (Lecia, Germany); Thermostatic oven and Water bath pot (Shanghai Jinhong); Upright microscope (Olympus, Germany); Fluorescence microscope (Olympus, Germany); Ultrasonic Cell crusher (Xinzhi, Ningbo); Tissue grinder and Metal bath (Eppendorf, Germany). Electrophoresis and Membrane transfer instrument (Bio-rad), Developing machine (Tanon).

### 2.4 Construction of aging mouse model

Sixty 8-week-old ICR female mice with normal estrous cycle were randomly divided into normal group (n = 10) and model group (n = 50) after adaptive feeding for 3 days. D-galactose powder was prepared into suspension with PBS buffer. According to the literature and previous pre-experimental results, the mice in the model group were given intraperitoneal injection of D-galactose suspension 125 mg/kg daily for 30 days, while the mice in the normal group were injected with the same dose of PBS buffer. Vaginal exfoliative cytological smears were performed for 14 consecutive days from the 16th day of modeling, which indicated that the aging mouse model was successfully constructed when the estrous cycle of most mice was disordered.

### 2.5 Animal grouping and medication

There were 10 mice in the normal control group (NC), and the mice in the model group were randomly divided into five groups, which were model control group (MC), low-, medium- and high-dose groups of Honghua Xiaoyao tablet (HHXYT-L, HHXYT-M, HHXYT-H) and Honghua Xiaoyao tablet medium-dose + estradiol group (HHXYT-M + E2). According to the table of “Equivalent Dose Ratio Converted by Body Surface Area between Humans and Animals”, HHXYT-L, M, H groups were given 0.3 g/kg, 0.6 g/kg and 1.2 g/kg HHXYT suspension by gavage, respectively, which is equivalent to 0.5, 1 and 2 times of the human dose. The dose in the HHXYT-M + E2 group was 0.6 g/kg Xiaoyao tablets +0.13 mg/kg estradiol valerate. The NC and MC were given the same amount of normal saline gavage, and the gavage was continued for 30 days starting on the 25th day of modeling.

### 2.6 Specimen collection

On the 16th day of gavage, the mice in each group underwent vaginal exfoliation cytology smears for 14 consecutive days. On the last night, the mice fasted but not restricted with water, and the samples were collected after anesthesia the next day. Before anesthesia, weigh and inject anesthesia intraperitoneally with 3.6% chloral hydrate solution, take blood from the orbit, stand at room temperature for 2 h, centrifuge at 4°C (3,000 rpm, 20min), take the upper serum to a new Ep tube, label it and store it at −20°C for testing. The mouse was placed on the operating table with the abdomen facing up, the abdominal cavity was dissected, the uterus and ovaries with excess adipose tissue removed were removed, weighed and recorded separately, and five ovaries were taken from each group and fixed in 4% paraformaldehyde solution. Store the rest of the tissue at −80°C.

### 2.7 Observe

#### 2.7.1 General condition and weight

The general state of the mice was observed daily, including appearance characteristics, mental state, diet, urine and feces conditions.

#### 2.7.2 Estrous cycle

At 9:00 a.m. every day, use a pipette gun to aspirate 10 μL PBS buffer, insert the tip of the pipette into the vagina of the mouse 2–3 mm, suction 3-5 times repeatedly. Collect vaginal exfoliated cells and coat them on the pre-labeled glass slide, dry them naturally and then carry out Wright-Gimesa composite staining solution staining. Observe the cell morphology under the microscope, and judge the estrous cycle in which they are located.

#### 2.7.3 Gonadal index

Ovarian index = wet weight of bilateral ovaries (g)/body mass of mice before death (g) × 100%, uterine index = wet weight of uterus (g)/body mass of mice before death (g) × 100%.

#### 2.7.4 ELISA method to determine serum sex hormone levels

In strict accordance with the instructions of the enzyme-linked immunoassay kit, the serum levels of FSH, LH, E2 and AMH in each group were detected. The absorbance value of each standard and sample was detected at a wavelength of 450 nm using a microplate reader. The concentration of each standard and the corresponding absorbance value were input into the ELISA Calc regression fitting calculation program to obtain a standard curve, and the sample absorbance value was substituted to calculate the sample concentration.

#### 2.7.5 Hematoxylin-eosin (HE) staining to observe ovarian morphology and follicle changes

After the ovarian tissue was fixed with 4% paraformaldehyde for 24 h, the ovarian tissue wax block was obtained by gradient ethanol dehydration, transparency, wax immersion, embedding, etc. The ovarian wax block was sectioned, bleached and selected with a thickness of 4 μm, and HE staining and sealing were carried out according to the operation steps, and the ovarian morphology of each group was observed under the microscope and photographed. The follicle count is carried out by two researchers, counting only follicles with oocyte nuclei, preventing the same follicle from being counted.

#### 2.7.6 Western blotting

Extract the protein from the ovarian tissue, and add the amount of protein lysate according to the weight of the tissue. SDS-PAGE was prepared with a lower layer of 10% separating gel, an upper layer of 5% stacking gel, electrophoresis, and the target protein was transferred to the PVDF membrane. Blots were incubated for 2 h in 5% non-fat milk in TBST. The blots were cut according to the required molecular mass, incubated with primary antibodies (1:1000) overnight at 4°C. The next day, the blots were rewarmed, washed, and incubated with secondary antibody (1:5000) for 2 h at moderate room temperature, developed with ECLplus reagent for imaging. ImageJ software quantitatively analyzed the gray values of different band proteins and calculated the relative expression levels.

#### 2.7.7 Immunohistochemistry (IHC)

The paraffin sections were made with HE staining, the paraffin sections were hydrated by gradient ethanol, 2% Tween permeabilization, citrate antigen retrieval at 96°C for 15 min, 3% hydrogen peroxide methanol to eliminate endogenous peroxidase activity, primary antibody was configured with 5% BSA at 1:50, incubated at 4°C overnight, secondary antibody was configured with PBS at 1:100, incubated at 37°C for 40 min. Then, slides were stained with DAB (1:20) and hematoxylin, followed by graduated ethanol treatment.

#### 2.7.8 Detection of apoptosis by TUNEL method

The paraffin sections were fully deparaffinized and hydrated, rinsed in PBS, and then permeabilized with 1× proteinase K working solution dropwise at 37°C, and rinsed again in PBS. The sample volume was calculated and the TdT labeling solution was centralized, and the samples were labeled after being treated with TdT equilibrium solution, and the reaction was carried out in a wet box for 60 min in the dark, and the samples were rinsed in PBS. The nuclei were counterstained with DAPI working solution dropwise and rinsed in PBS. The sections were covered with antifluorescence quencher and the results were observed under a fluorescence microscope.

### 2.8 Statistical analysis

Experimental data were expressed by mean ± standard error (SEM), and independent sample nonparametrical test (Kruskal–Wallis rank-sum test) was used for data analysis between groups. *p* < 0.05 was statistically significant. Data were analyzed using SPSS 24.0 and graphed using GraphPad Prism 8.0 and Photoshop.

## 3 Results

### 3.1 Effect of HHXYT combined with estradiol on gonadal index in aging mice

**FIGURE 1:**
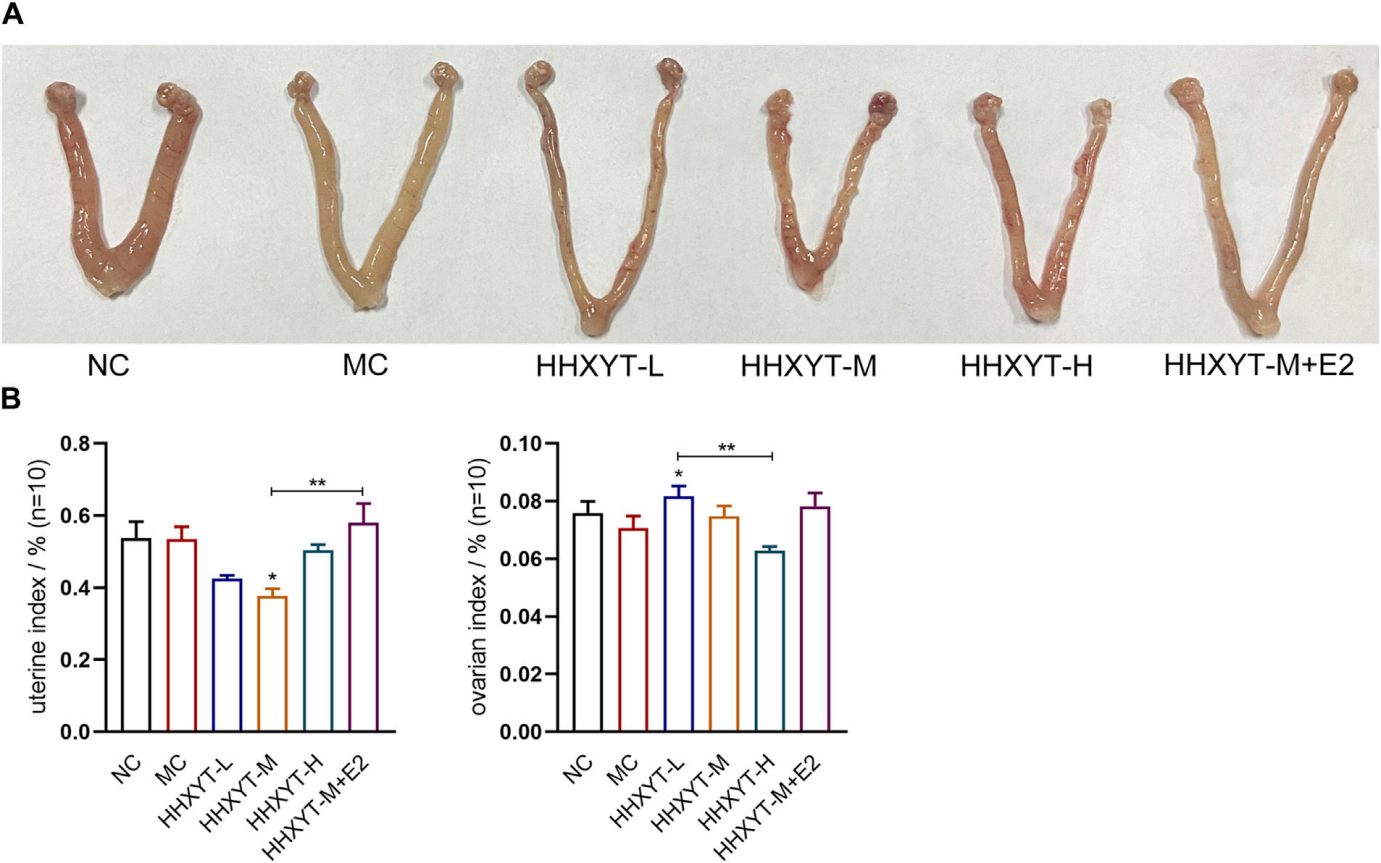
Effects of HHXYT combined with estradiol on genital appearance and gonadal index in aging mice. **(A)** Appearance of the uterus and ovaries of mice in each group at the end of the experiment. **(B)** Weigh with electronic scale and calculate the weight of uterus or bilateral ovaries (G)/mouse weight (G). The data presented as mean ± SEM. Compared to the model control group: *p < 0.05, **p < 0.01.

There was no significant difference in appearance and posture among the groups. The changes in the morphology of the ovaries and uterus of each group are shown (Figure 1A). There was no significant change in the uterine index in the MC group. The uterine index decreased in all dose groups compared to the MC group (HHXYT-M vs. MC: *p* < 0.05), and increased in the HHXYT-M + E2 group. Compared with the NC group, the ovarian index in the MC group decreased slightly. Compared with the MC group, the ovarian index reduced in the high-dose group and raised in the low-dose group and the HHXYT-M + E2 group (HHXYT-L vs. MC: *p* < 0.05) (Figure 1B).

### 3.2 Effect of HHXYT combined with estradiol on the estrous cycle of aging mice

**FIGURE 2:**
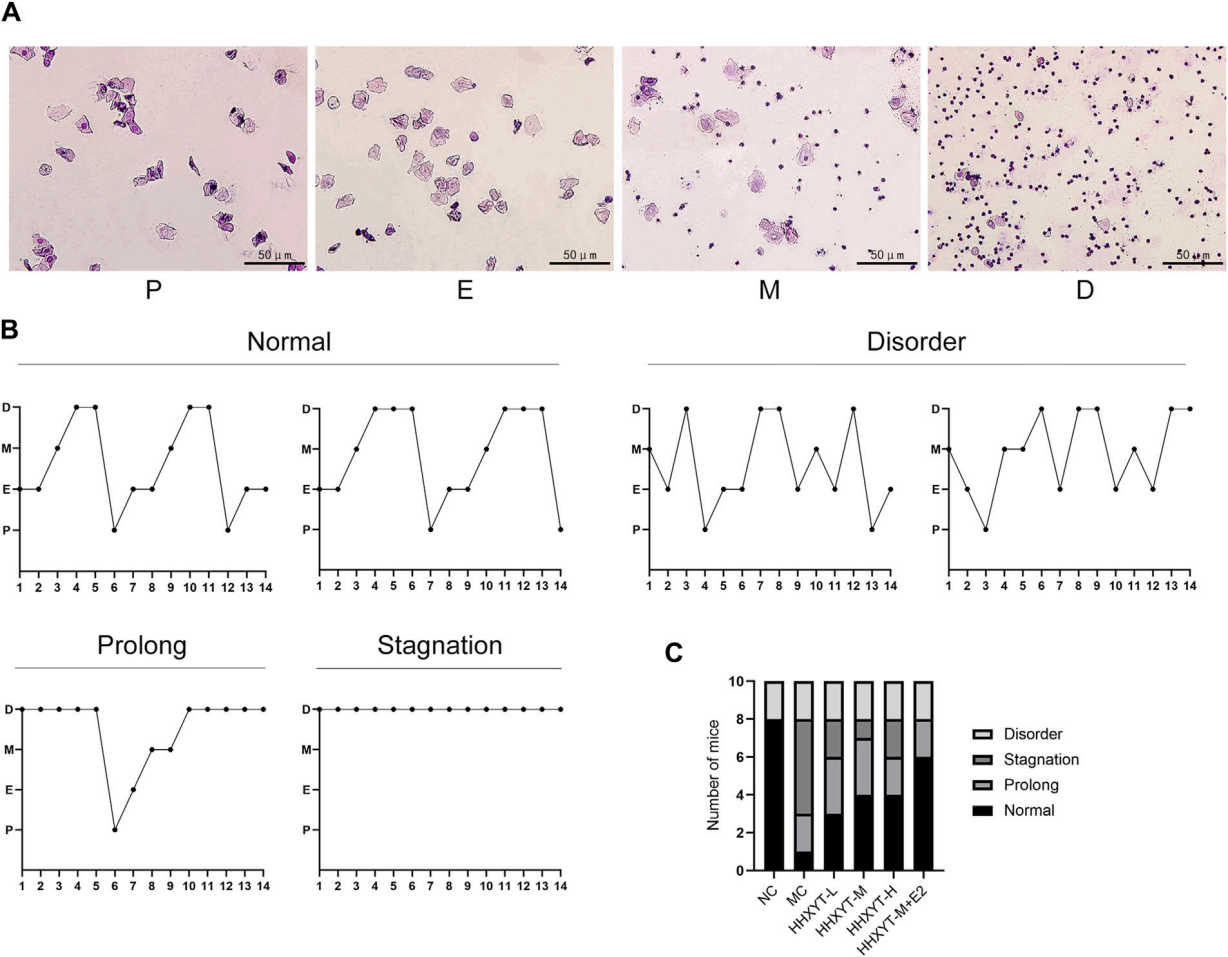
Effect of HHXYT combined with estradiol on the estrous cycle in aging mice. **(A)** The four typical pictures of exfoliated cells stained with Wright-Gimsa solution represent the proestrus (P), estrus (E), metestrus (M), and diestrus (D) stages in the estrus cycle. The scale bar represents 50 μm. **(B)** Common patterns of regular and irregular estrus cycles. **(C)** Number of mice with different modes of estrous cycle in each group.

The morphological characteristics of each stage of the estrous cycle are as follows: Proestrus (P): a large number of nucleated epithelial cells are dominant; Estrus (E): predominantly large, flattened, irradiated margins of nucleated keratinized squamous epithelial cells, without leukocytes; Metestrus (M): decreased keratinizing epithelial cells and nucleated epithelial cells and leukocytes; Diestrus (D): predominantly a large number of white blood cells. Through continuous observation of vaginal exfoliated cells, it was found that the estrous cycle of normal ICR female mice was about 5–6 days, which appeared according to the above estrous cycle rules. Wright-Giemsa staining results from mouse vaginal exfoliated cell smears are shown (Figure 2A). Common estrous cycle patterns in experiments are shown (Figure 2B). The estrous cycles of mice in the NC group were regular. The estrous cycles in the MC group were prolonged, stagnant or had no obvious cycle. The estrous cycles in the HHXYT group recovered to varying degrees, and the recovery effect in the HHXYT-M + E2 group was more obvious (Figure 2C).

### 3.3 Effects of HHXYT combined with estradiol on serum sex hormone levels and FSHR expression in ovarian tissue in aging mice

**FIGURE 3:**
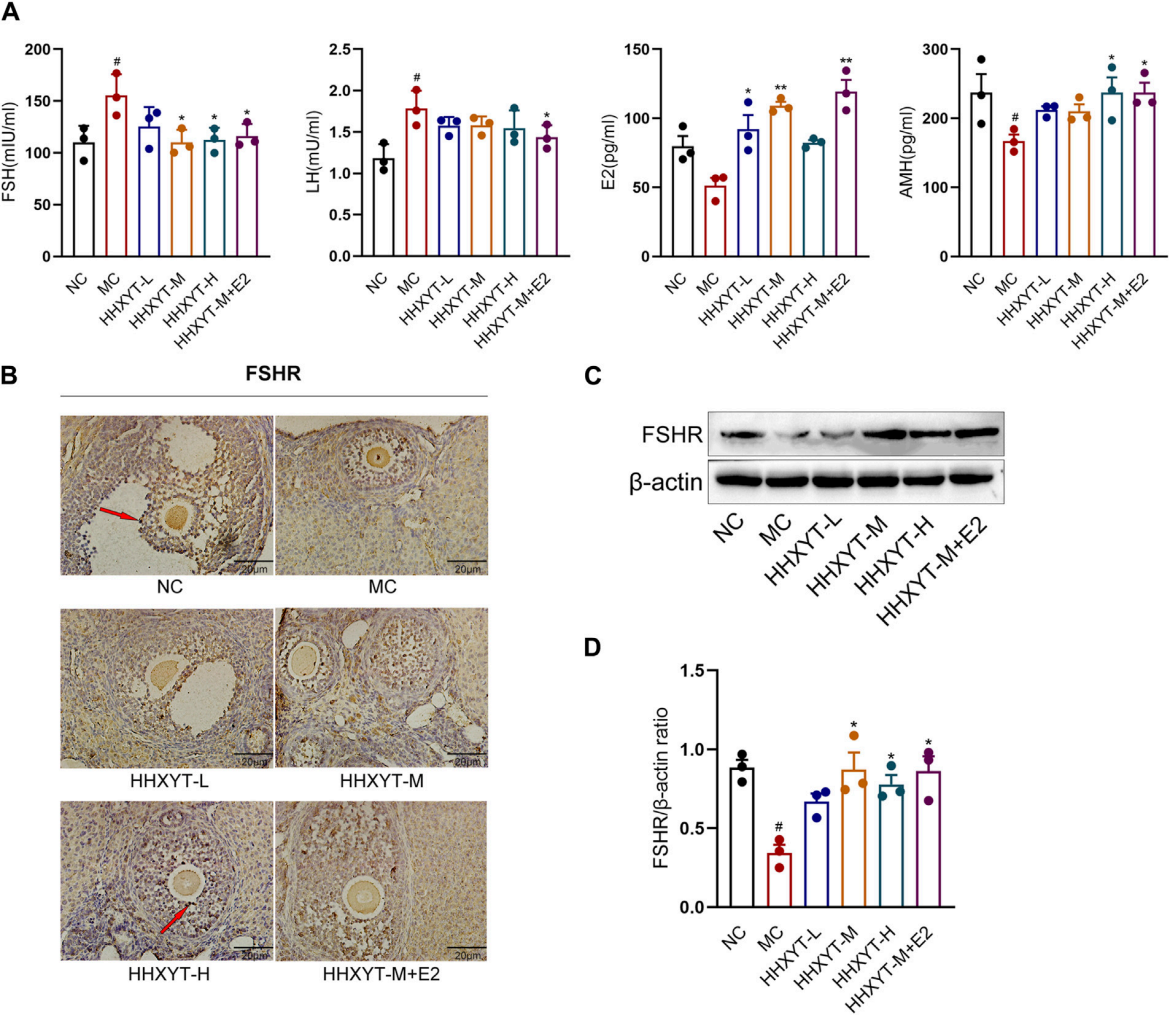
Effect of HHXYT combined with estradiol on serum hormonal levels and ovarian FSHR expression in aging mice. **(A)** The serum levels of FSH, LH, AMH and E2 were measured by ELISA. **(B)** The expression and localization of FSHR in mouse ovary were detected by IHC. The dark stain indicated by the red arrow is positive for cells. The scale represents 20 μm. **(C)** The expression of FSHR in mouse ovary were detected by Western blot. **(D)** The gray value of FSHR protein expression in ovarian tissue of mice in each group was statistically analyzed. Compared to the normal control group: #*p* < 0.05; Compared to the model control group: *p < 0.05, **p < 0.01.

Compared with the NC group, the serum levels of FSH and LH in the MC group increased significantly (FSH, LH: *p* < 0.05), and the levels of E2 and AMH decreased (AMH: *p* < 0.05). Compared with the MC group, the FSH level reduced to varying degrees, and the FSH level decreased significantly in the medium- and high-dose groups and the HHHXYT-M + E2 group (HHXYT-M + E2 vs. MC: *p* < 0.05). The LH level decreased in each dose group of HHXYT, but there was no significant difference between groups. Compared with the MC group, the LH level in the HHXYT-M + E2 group reduced significantly (HHXYT-M + E2 vs. MC: *p* < 0.05). The E2 level increased to different degrees, and the HHXYT-M + E2 group raised significantly (HHXYT-M + E2 vs. MC: *p* < 0.01). With the increase of the dose of HHXYT, the level of AMH increased slightly, and the combined E2 group raised significantly (HHXYT-M + E2 vs. MC: *p* < 0.05) (Figure 3A).

Western blot and immunohistochemistry were used to detect the expression of FSHR in ovarian tissues, and the results showed that the expression of FSHR in the MC group was significantly reduced compared with the NC group (*p* < 0.05). Compared with the MC group, the expression level of FSHR in each dose group of HHXYT increased differently (HHXYT-M vs. MC: *p* < 0.05), and there was no significant change in HHXYT-M + E2 compared with the HHXYT-M alone (Figures 3B–D).

### 3.4 Effects of HHXYT combined with estradiol on ovarian morphology and follicle count in aging mice

**FIGURE 4:**
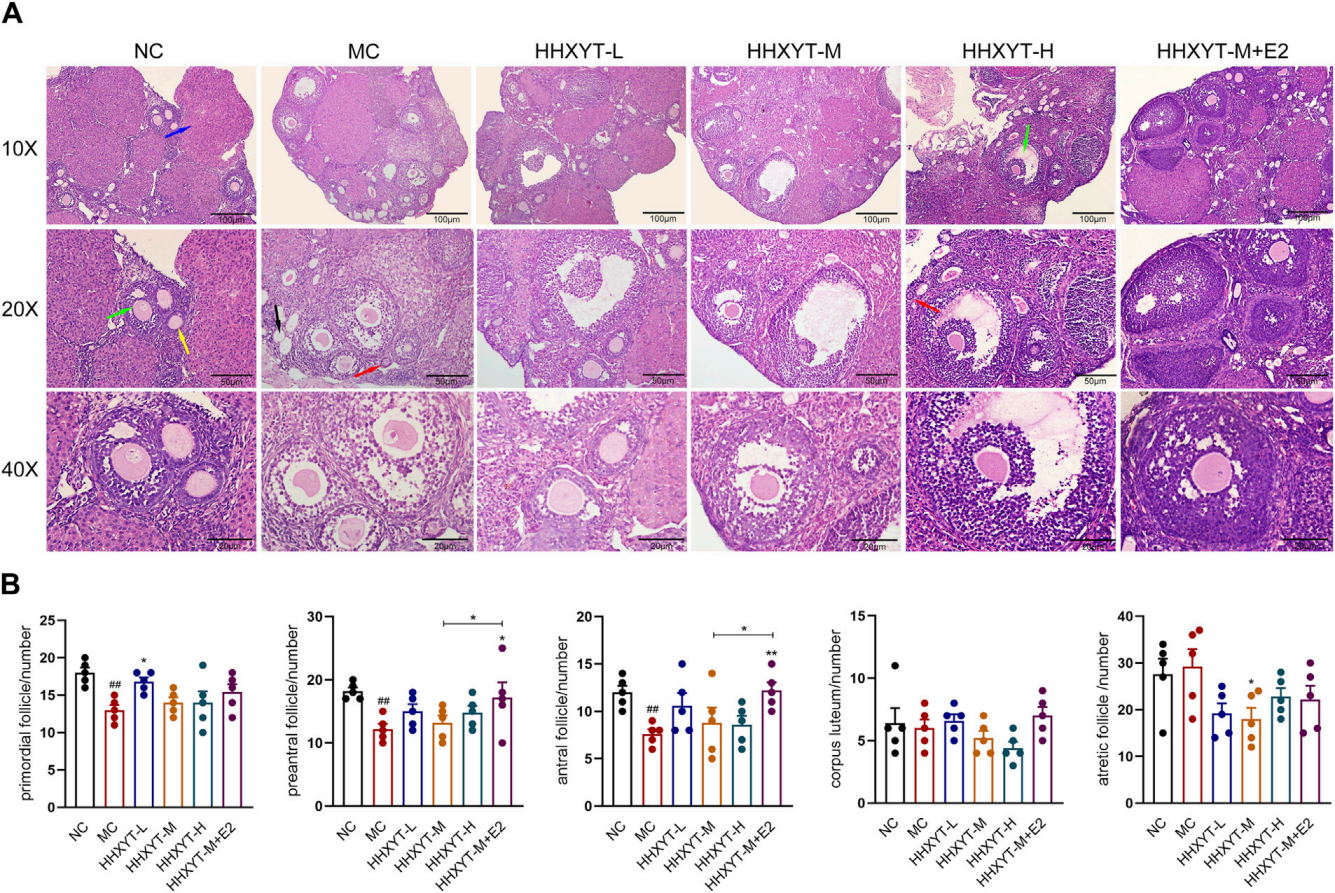
Effect of HHXYT combined with estradiol on ovarian morphology and follicle count in aging mice. **(A)** The ovarian tissue was stained with H&E and photographed under a microscope at ×10, ×20, ×40 magnification. The scale bar represents 100, 50, 20 μm respectively. The red, yellow, green, blue, and black arrows represent the primordial follicles, preantral follicles, antral follicles, corpus luteum, and atresia follicles, respectively. **(B)** The primordial, preantral, antral, corpus luteum and atretic follicles were counted. Compared to the normal control group: ##*p* < 0.01; Compared to the model control group: *p < 0.05, **p < 0.01.

The ovarian morphology and follicle count of the mice in each group are shown (Figure 4A). Under the microscope, it can be observed that the number of follicles at all levels in the NC group was more, the morphology was normal, and the granulosa cells were neatly arranged. In the MC group, the number of granulosa cells decreased, the arrangement was loose and disordered, the number of growing follicles decreased, and the atretic follicles had no significant change. Compared with the MC group, the number of primordial follicles in the low-dose group increased (HHXYT-L vs. MC: *p* < 0.05), while in the other groups had no statistical significance. After intervention with HHXYT, the number of atretic follicles reduced (HHXYT-M vs. MC: *p* < 0.05). There was no significant change in the number of corpus luteum among groups. HHXYT + E2 raised the number of preantral follicles and antral follicles, and the effect was more obvious than that of HHXYT alone (Figure 4B). What’s more, the number of layers and density of granulosa cells increased significantly after combining estradiol.

### 3.5 Effect of HHXYT combined with estradiol on the expression of apoptosis-related signaling molecules in ovarian tissues of aging mice

**FIGURE 5:**
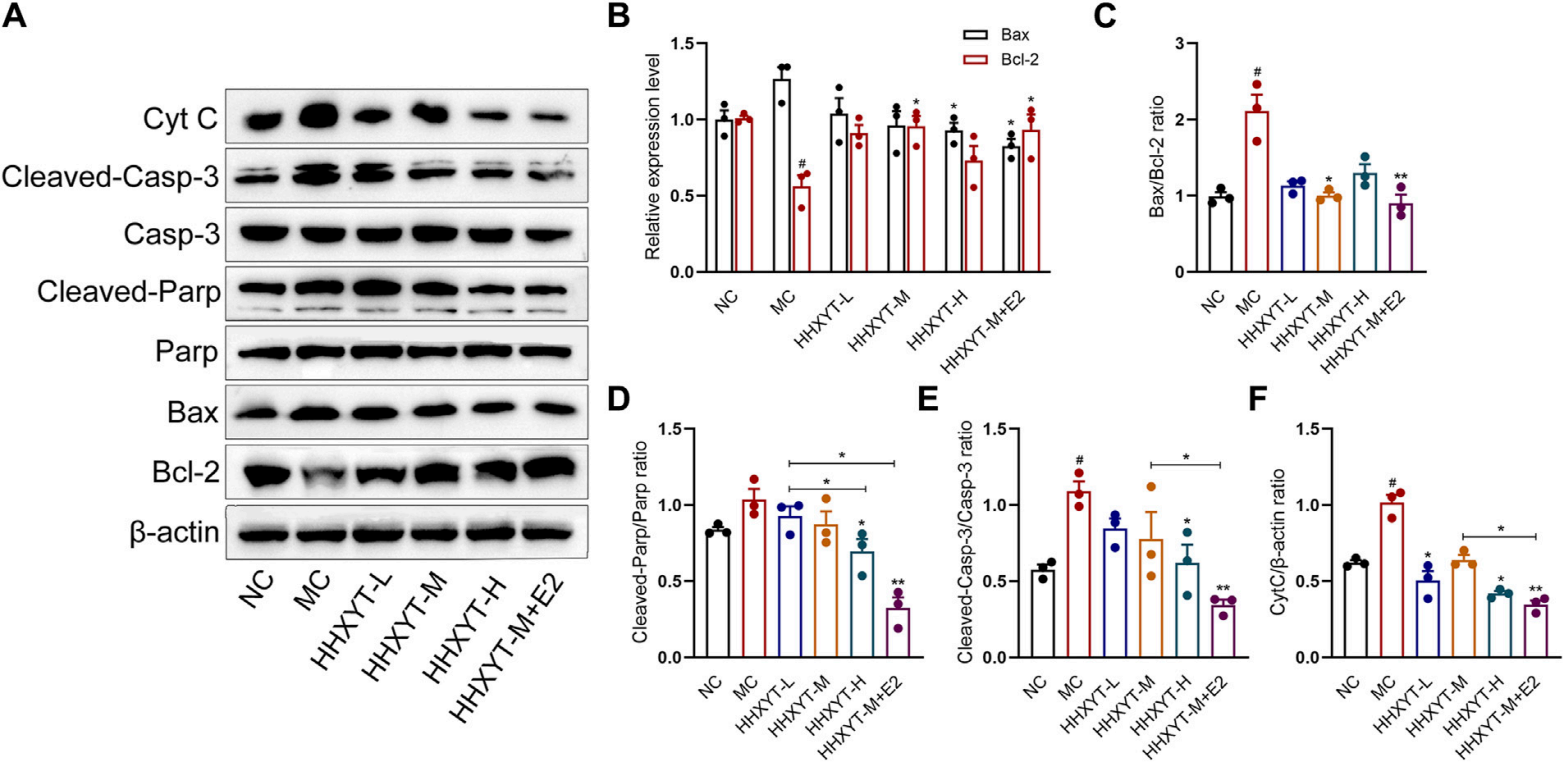
Effect of HHXYT combined with estradiol on protein expressions of Bax, Bcl-2, Parp, cleaved-Parp, Casp-3, cleaved-Casp-3 and CytC in aging mice. **(A)** The levels of Bax, Bcl-2, Parp, cleaved-Parp, Casp-3, cleaved-Casp-3 and CytC in mouse ovarian tissues were detected by Western blot. The relative gray values of Bax, Bcl-2 **(B)**, Bax/Bcl-2 **(C)**, cleaved-Parp **(D)**, cleaved-Casp-3 **(E)** and CytC **(F)** proteins in ovarian tissues of mice in each group were statistically analyzed. Compared to the normal control group: #*p* < 0.05; Compared to the model control group: *p < 0.05, **p < 0.01.

Compared with the NC group, the expression of Bcl-2 protein in the MC group decreased, the expression of Bax protein increased, and the Bax/Bcl-2 ratio was twice that of normal mice (*p* < 0.05). The relative expression of CytC protein raised significantly (*p* < 0.05). Compared with the MC group, the expression of Bcl-2 increased to varying degrees, and the level of Bax gradually reduced with the augment of dosage. The expression effect of combined estradiol group was better than that of the single HHXYT group (Bax/Bcl-2: HHXYT-M + E2 vs. MC: *p* < 0.01). The expression of CytC protein decreased significantly in the combined estradiol group (HHXYT-M + E2 vs. MC: *p* < 0.01) (Figures 5A, B, C, F).

There was no significant change in the total protein expression levels of Parp and Casp-3 among the groups. Compared with the NC group, the expression of cleaved-Parp and cleaved-Casp-3 in the MC group raised (cleaved-Casp-3: *p* < 0.05). Compared with the MC group, the expression of cleaved-Parp and cleaved-Casp-3 gradually reduced with the augment of dosage, and the protein expression decreased most significantly in the combined estradiol group (both HHXYT-M + E2 vs. MC: *p* < 0.01) (Figures 5A, D, E).

### 3.6 Effect of HHXYT combined with estradiol reduce apoptosis of granulosa cells in ovarian tissues of aging mice

**FIGURE 6:**
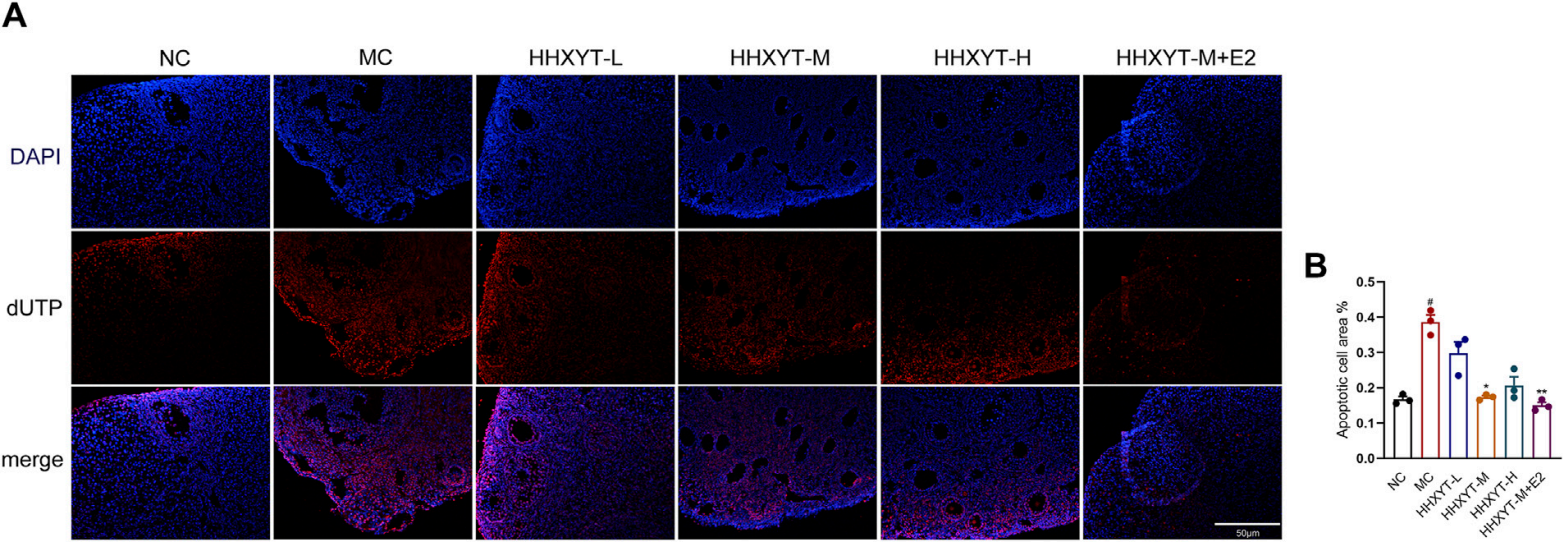
Effect of HHXYT combined with estradiol on apoptosis of ovarian granulosa cells in aging mice. **(A)** Apoptosis was analyzed by *in situ* TUNEL fluorescence assay (20×). In the TUNEL assay, nuclei of TUNEL-positive (apoptotic) cells are red. The scale represents 50 μm. **(B)** The number of TUNEL-positive granulosa cells between the groups was compared. Compared to the normal control group: #*p* < 0.05; Compared to the model control group: *p < 0.05, **p < 0.01.

We observed red-stained TUNEL positive (apoptosis) nuclei in the growing follicles, but not in the primordial follicles. D-galactose increased the number and area of apoptotic cells (*p* < 0.05). Compared with the MC group, the number of apoptotic cells in the middle-dose group was reduced, but significantly decreased in the HHXYT-M + E2 group (HHXYT-M + E2 vs. MC: *p* < 0.01) (Figure 6).

## 4 Discussion

D-galactose is an aldehyde hexanese, which is a reducing sugar naturally present in the body and in many foods, and a moderate amount of galactose can be metabolized and eliminated within a certain period of time. At high levels, it can be converted to aldose and hydrogen peroxide catalyzed by galactose oxidase, resulting in reactive oxygen species (ROS). Increased ROS leads to cell damage, which can lead to aging of organisms (Davalli et al., 2016). All available data suggest that the decline in ovarian function with age is primarily due to oxidative stress (Broekmans et al., 2009; Wang et al., 2020). Taking D-galactose causes several changes in the reproductive system, similar to the aging process. In animals treated with D-galactose, estrogen levels decreased and serum LH and FSH levels increased, comparable to those in the natural aging group, while disrupting the estrous cycle and damaging uterine and ovarian tissue (Ahangarpour et al., 2016). In our experiments, the estrous cycle of D-galactose-induced aging mice was disrupted, with significant increases in serum FSH and LH, and decreased levels of E2 and AMH, which is consistent with previous studies. HHXYT may have the effect of improving ovarian function in several studies (Hui-Fang, 2014; Wu et al., 2022). In this experiment, HHXYT combined with estradiol could improve the estrous cycle of aging mice, increase serum E2 and AMH levels, and reduce FSH and LH levels.

From the pathological point of view, galactose targets different stages of follicle development, and follicles affected by exposure to galactose at different times before and after birth are different (Rostami Dovom et al., 2019). Ovarian aging is characterized by a decrease in the number of follicles and a decrease in oocyte quality (Chon et al., 2021). We used D-galactose-induced senescence in mice, which mainly affected the occurrence and development of primordial follicles and growth follicles at all levels, and the number of atresia follicles increased slightly. After the treatment with low and medium doses of HHXYT, the number of atresia follicles decreased. The combination of HHXYT and estradiol increased the number of preantral follicles and antral follicles, and the effect was more obvious than that of HHXYT alone. Microscopy of aging mice showed that the number of granulosa cells was reduced and loosely arranged. The number and density of granulosa cells increased after the combination of HHXYT combined with estradiol, and the effect was significant. Granulocytes (GCs) are essential for follicular development and homeostasis, and they provide nutritional and mechanical support to oocytes through physical interactions (Rimon-Dahari et al., 2016). Observing the process of follicles from growth to atresia, it was found that the number of apoptosis of GCs was never found to gradually increased, suggesting that apoptosis of GCs often leads to follicular atresia and ovarian aging (Matsuda et al., 2012). We showed an increase in the number of granulosa apoptosis in the growing oocytes of senescent mice induced by D-galactose. The number of apoptosis of granulosa cells was significantly reduced after treatment with HHXYT combined with estradiol. Combined with the above results, we concluded that compared with HHXYT alone, HHXYT combined with estradiol could better improve the number and quality of follicles.

Oxidative stress in the ovarian microenvironment is a major driver of the ovarian aging process and promotes the development of ovarian aging-related etiologies such as apoptosis, inflammation, and mitochondrial dysfunction (Yang et al., 2020). Apoptosis has been extensively studied in ovarian aging (Shen et al., 2019). Granulotic apoptosis, which can lead to extensive follicular atresia or degeneration, is considered one of the most important mechanisms leading to ovarian aging (Regan et al., 2018; Yang et al., 2019). The ratio of intracellular pro-apoptotic genes (e.g., expression of Bax) to inhibitory genes (e.g., expression of Bcl-2) determines whether cells will undergo apoptosis or survival (Tripathi et al., 2013; Chaube et al., 2014). Oxidative stress induces ovarian apoptosis through a variety of pathways, including the exogenous (death receptor) pathway and the endogenous (mitochondria) pathway (Yadav et al., 2018). First, excess oxidative stress can induce activation of the mitochondrial pathway, leading to a reduced ability of mitochondria to produce ATP, altering the membrane potential by regulating the Bax/Bcl-2 ratio (Jurisicova and Acton, 2004; Tiwari et al., 2017), resulting in the release of CytC from the mitochondria into the cytosolium (Tripathi et al., 2013). Cytochrome C binds to the apoptotic proteolytic enzyme activator (APAF-1) and continues to activate Caspase-9 (Monian and Jiang, 2012; Xiong et al., 2014). Activated Caspase-9 triggers an active shift in Casp-3 (Tripathi et al., 2012). The effector Casp-3 performs the final step of apoptosis in female germ cells, lysing structural and regulatory proteins in oocytes, resulting in an apoptotic profile on cell morphology (Xiong et al., 2014). Several studies have shown that the activation of Bax protein and Casp-3 is involved in oocyte apoptosis in rat and mouse oocytes (Chaube et al., 2005a; b; Teixeira et al., 2019). One of the important substrates that Casp-3 can cleave is Parp, which is a key enzyme for detecting DNA damage and occurrence (Decker and Muller, 2002; Liu et al., 2016). The presence of cleaved-Parp is one of the most commonly used diagnostic tools for detecting apoptosis in a variety of cell types (Bressenot et al., 2009). The expression of apoptosis-related proteins was analyzed by Western blot. The ratio of Bax/Bcl-2 in the ovarian tissues of D-galactose mice was higher than that of normal mice, and the expression of CytC protein was significantly increased, and the expressions of cleaved-Casp-3 and cleaved-Parp were also increased. After the treatment of HHXYT, the expression of Bcl-2 increased to varying degrees, and the level of Bax gradually reduced with the augment of dosage, and the expression of cleaved-Parp and cleaved-Casp-3 gradually decreased. The expression of CytC protein decreased particularly in the HHXYT-M + E2 group, and in general, the decrease in the expression of pro-apoptotic molecules in the combined estradiol group was better than that in the HHXYT alone group. This mechanism of HHXYT may be related to certain anti-inflammatory and antioxidant components in the drug.

In addition, we analyzed the expression of FSH receptor in ovarian tissue. FSH promotes follicle growth by acting on FSHR, which is mainly expressed on granulosa cells within the follicle, tightly controlling follicular development in response to periodic pituitary FSH secretion (Simoni et al., 1997; Lazaros et al., 2012). The expression of FSHR in the model group decreased, and the FSHR level in the HHXYT-M,H group and the HHXYT-M + E2 group increased, which was opposite to the expression level of FSH. This expression of FSHR may be related to granulosa cell apoptosis and positive and negative feedback regulation of the pituitary-gonadal axis.

## 5 Conclusion

Through this study, we found that the combination of HHXYT and estradiol could improve the estrous cycle, serum sex hormone levels and the number of growing follicles at all levels in D-galactose-induced aging mice, and significantly increased the number and layers of granulosa cells. The mechanism may be to regulate the occurrence of apoptosis by reducing the apoptosis of ovarian granulosa cells and affecting the expression ratio of Bax/Bcl-2. In short, HHXYT alone has a certain effect on improving ovarian function, but the combination of estradiol may have a better therapeutic effect. This study provides some reference for clinical related fields and drug development.

## Data Availability

The raw data supporting the conclusion of this article will be made available by the authors, without undue reservation.
